# Fatigue and Affective Manifestations in Multiple Sclerosis—A Cluster Approach

**DOI:** 10.3390/brainsci10010010

**Published:** 2019-12-22

**Authors:** Samar S. Ayache, Moussa A. Chalah

**Affiliations:** 1 Service de Physiologie, Explorations Fonctionnelles, Hôpital Henri-Mondor, AP-HP, 94010 Créteil, France; samarayache@gmail.com; 2 EA 4391, Excitabilité Nerveuse et Thérapeutique, Université Paris-Est-Créteil, 94010 Créteil, France

**Keywords:** multiple sclerosis, MS, depression, anxiety, fatigue, tDCS

## 1. Introduction

Multiple sclerosis (MS) is a chronic autoimmune disease of the central nervous system, characterized by a high prevalence in young people, a drastic impact on the quality of life, and an important economic cost to society. Throughout the disease course, MS patients suffer from a plethora of symptoms that could seriously alter their performance at work, affect their relationships with peers and families, and be at the origin of unemployment and/or divorce. Among these symptoms, fatigue stands as one of the most common complaints and is usually perceived as the most debilitating MS-related problem. Indeed, 75%–90% of MS patients suffer from fatigue at some point during their lifetime and could be left without an efficient solution despite the tremendous effort usually exerted by the medical staff [1].

The scientific and medical community considers MS fatigue a complex and multifaceted symptom. In fact, its definition remained vague for a long period of time until the MS council for clinical practice guidelines set a clear description of this complaint and has defined it as ‘a subjective lack of physical and/or mental energy that is perceived by the individual or caregiver to interfere with usual and desired activities’ [2]. Hence, fatigue reported by MS patients differ from the ‘classical’ tiredness experienced by healthy individuals in that it seriously impedes daily activities and can be lessened or heightened by cold or hot weather, respectively [2]. It is also important to highlight the multidimensional aspect of this symptom. Indeed, it is now widely accepted that MS fatigue implies three components: physical, psychosocial, and cognitive. All of the components are screened for and assessed in a subjective manner, using some available questionnaires, among which, we can cite the modified fatigue impact scale, a 21-item tool that permits clinicians to evaluate the three dimensions of fatigue [1].

From a pathophysiological standpoint, MS-related abnormalities have been incriminated in the generation of fatigue and have been largely studied and described over the past decade. Other factors seem to be implicated, such as inflammatory mediators (i.e., TNF-α, IFN-γ, and IL-1β), neuro-immune and neuro-endocrine mechanisms (i.e., hypothalamo-pituitary-adrenal axis) [2,3].

In addition to fatigue, affective symptoms are particularly worrisome in this population. The last century has witnessed a growing literature on the existence of a potential link between MS and psychiatric manifestations [4]. Among these manifestations, anxiety and depression are of particular interest, could respectively affect 41%–50% of MS patients, and would be responsible for a deep suffering [5]. Compared to the general population, lifetime prevalence of major depressive disorders, anxiety disorders, and suicidal attempts are higher in MS patients (rates being double those reported in the general population).

Although one might perceive anxiety and depression as logical consequences of, or pure reactions to, a chronic debilitating illness, this perception should be adopted with caution and could not explain by itself the occurrence of these symptoms. Several reports have documented the existence of psychiatric relapses in the context of MS [5], pointing towards the presence of destructive lesions behind the emergence of these symptoms. Apart from the acute onset of depressive or anxious episodes, pathological changes (i.e., demyelinating lesions and axonal generation) usually accumulate along the disease course and might contribute to an insidious development and progressive aggravation of these affective manifestations. This viewpoint has been clearly expressed in the Golden consensus statement on depression in MS [6]. This statement published in 2005, highlighted the involvement of MS immunopathology in the generation of affective disorders and stressed on the importance of good screening and efficacious management of these troubles.

Interestingly, fatigue, anxiety, and depression tend to coexist in this clinical population, and the concept of a symptoms cluster has been recently introduced, pointing towards a potential interaction among them and the existence of common underlying mechanisms [7,8,9,10,11,12].

In the following sections, we will address the possible relationship that seems to exist among these symptoms and the common mechanisms that might underlie them. Then, we will briefly discuss some of the available therapeutic strategies.

## 2. Bidirectional Relationship

As stated above, a close link seems to exist between fatigue, anxiety, and depression and could be illustrated by the cognitive and behavioral model of MS fatigue which involves an interaction between cognitions, emotions, behaviors, and biology [13]. In fact, fatigue perception, per se, may render the person more anxious and worried about their health condition [12]; they may have some fears about re-experiencing fatigue in particular and about their future in general. This may lead to aberrant behaviors (e.g., avoiding/resting behavior and a restriction of participation in psychosocial activities), which can subsequently cause a state of unhappiness, melancholy, and anxiety. This would on its turn reinforce the feeling of fatigue and demotivation. Thus, a vicious circle would set in and becomes difficult to manage.

## 3. Common Pathophysiological Mechanisms

In fact, from a pathophysiological viewpoint, anxiety and depression in MS were associated in some works with pathologies involving the frontal lobes and/or their connections [14,15,16]. Temporal, parietal, and limbic abnormalities have also been incriminated in the generation of depression [17,18,19,20]. Moreover, a link has been recently established between septo-fornical damage and the development of anxious manifestations in this population [11].

As for MS fatigue, the neural substrates suggested to be at the basis of this symptom are yet to be fully clarified. However, recent advances in neuroradiological imaging techniques have provided researchers with some invaluable data as to where the putative lesions are located. A huge number of studies have been conducted to assess the potential pathophysiological processes of this complaint. Although, at a first glance, discrepancy seems to exist across the studies regarding the nature of the reported abnormalities (e.g., cortical atrophy, deep gray matter atrophy, white matter lesions) and their locations; these data are in reality complementary, and when taken together, they mostly involve a large cortico-thalamo-striato-cortical loop. Such a loop implies numerous cortical and subcortical regions, among which the fronto-parietal areas and their connections seem to be highly implicated [2,21].

By adopting a more global vision, one may consider that fatigue, anxiety, and depression, share common pathophysiological mechanisms. In fact, MS lesions would alter numerous cortico-cortical and cortico-subcortical connections, and result in what is known as the “multiple disconnection syndrome” [22,23]. When these disconnections are clustered in specific locations, they would lead to specific symptoms as we have previously illustrated. In other words, each symptom relies on a network made of hubs and connections. When lesions destroy a key hub, it would lead to the generation of a specific symptom. Given the fact that certain hubs are common for several symptoms, damage of these hubs would lead to a cluster of complaints, thus explaining the cooccurrence of fatigue, depression, and anxiety in MS patients (e.g., correlation between forceps minor damage and co-occurrence of fatigue and depression as reported by Gobbi and colleagues [15]).

## 4. Therapeutic Approach and Perspectives

Concerning depression and anxiety, their management follows what is recommended in other clinical populations. It usually consists of antidepressants and anxiolytics combined or not with psychotherapy [24]. Efficacy of these approaches has been proven in the general population with evidence supporting their utility in patients without MS. However, in patients with MS, recommendations are lacking, and little is known as to which adequate molecule and/or the most appropriate psychotherapeutic modality to choose in front of an emotional disorder in this context [24].

Despite the available pharmacological options, some patients might suffer from resistant depression. The latter has been recently reported in a series of MS patients, in whom electroconvulsive therapy was tried and some efficacy has been shown [25]. However, the safety of this intervention in the setting of MS has not been elucidated yet [25].

Regarding fatigue, the available pharmacological strategies are limited to few treatments, most of which ended up being inefficient or responsible for several annoying side effects [1]. The fact that most of the available treatments are unsatisfactory makes the management of MS fatigue disappointing in the majority of cases. Facing this reality, alternative therapeutic approaches are highly required. To fulfill this need, some researchers have been particularly interested in testing the efficacy of transcranial direct current stimulation (tDCS)—a noninvasive tool—in the management of MS-related symptoms [26,27]. The safety profile of tDCS has been widely documented, however its place in the therapeutic arsenal of these symptoms is yet to be defined.

MS lesions affecting frontal lobes and their connections have been found to underly the emergence of fatigue, depression, and anxiety. Interestingly, depression studies have shown that the application of an anodal tDCS over the dorsolateral prefrontal cortex (DLPFC) would be beneficial in treating major depressive disorders. In the setting of MS, few reports have shown promising effects of the left anodal prefrontal tDCS. Indeed, this intervention was found in some studies to significantly decrease fatigue severity when applied over the left DLPFC for 20 min [28]. Such antifatigue effects were only obtained when the sessions were repeated daily for five consecutive days [1,28]. Since tDCS effects seems transient and could fade away one to two weeks later, some case studies aimed to prolong the efficacy and have documented that repetition of stimulation sessions could guarantee the maintenance of such effects [29,30], and could significantly decrease the anxiety and depression scores, the latter being evaluated as secondary endpoints. Hence, tDCS might have a place in the antifatigue armamentarium and possibly in the management of affective symptoms in MS patients.

However, repeating tDCS sessions could represent a real obstacle in clinical practice. This implies daily or weekly visits to the hospital and can represent a real burden for this population already tired by the illness and its related physical handicap. In order to overcome such a problem, delivering tDCS at home seems to be an optimal solution. Feasibility and safety of home-based tDCS has been recently proven and its ability to ameliorate fatigue has been also shown by the same team [31,32]. Future works are obviously needed to further test this modality, present an in-depth assessment of its impact on this interesting cluster of symptoms, and provide the medical community with an adequate scheme to follow each time the opportunity arises.
